# Machine Learning Algorithms to Predict Heavy Episodic Drinking in the United States Using Survey Data

**DOI:** 10.1111/dar.70065

**Published:** 2025-11-04

**Authors:** Laura Llamosas‐Falcón, Charlotte Probst, Kevin Shield, Erik Spence, Jürgen Rehm

**Affiliations:** ^1^ Institute for Mental Health Policy Research Centre for Addiction and Mental Health Toronto Canada; ^2^ Institute of Medical Science Faculty of Medicine, University of Toronto, Medical Sciences Building Toronto Canada; ^3^ Campbell Family Mental Health Research Institute Centre for Addiction and Mental Health Toronto Canada; ^4^ Department of Psychiatry Faculty of Medicine, University of Toronto Toronto Canada; ^5^ Dalla Lana School of Public Health University of Toronto Toronto Canada; ^6^ SciNet Consortium University of Toronto Toronto Canada; ^7^ Center for Interdisciplinary Addiction Research, Department of Psychiatry and Psychotherapy University Medical Center Hamburg‐Eppendorf Hamburg Germany; ^8^ Program on Substance Abuse & WHO European Region Collaboration Centre Public Health Agency of Catalonia Barcelona Spain

**Keywords:** alcohol use, heavy episodic drinking, machine learning, public health, SHAP

## Abstract

**Introduction:**

Heavy episodic drinking (HED) is a major public health concern but is often missing from surveys or measured unreliably. Predictive models offer a method to estimate HED's likelihood at the individual level in such cases. While logistic regression is commonly used, other machine learning algorithms (MLA) may offer greater accuracy and robustness. This study compares various MLAs to identify the best predictive model of HED.

**Methods:**

Data from the 1997–2018 National Health Interview Survey were used. Six MLAs were trained and cross‐validated: logistic regression, naïve bayes, k‐nearest neighbour, support vector machine, random forest and XGBoost. Model performance was compared, and the SHapley Additive exPlanations (SHAP) method assessed interpretability by ranking features based on their contribution to the model's prediction.

**Results:**

The probability of correctly ranking a randomly selected HED instance higher than a non‐HED instance ranged from 0.85 to 0.97 (with values closer to 1 indicating better performance). XGBoost outperformed the other MLAs (sensitivity 0.80, precision 0.83, accuracy 0.92). Amongst the 11 features included in the models, average daily alcohol use and age were the most influential, as determined by SHAP values.

**Discussion and Conclusions:**

The strong discriminative ability of our models shows that even a limited number of well‐chosen features can yield robust predictions, highlighting the potential of MLAs for modelling health behaviours. Integrating our models into simulation frameworks can help model HED and test scenarios, leading to effective policies. Future studies should incorporate objective sources for external validation and investigate systematic biases to improve predictive accuracy.

**Summary**

- Amongst the six machine learning algorithms tested, the probability of correctly ranking a heavy episodic drinking instance ranged from 0.85 to 0.97, with XGBoost outperforming the other models.
- Average daily alcohol use and age were identified as the most impactful features influencing the model's predictions of heavy episodic drinking.
- Our findings underscore an opportunity for a shift towards more advanced modelling approaches in alcohol research.

## 1 Introduction

Heavy episodic drinking (HED), defined as consuming a large amount of alcohol in a short period, typically five or more drinks for males and four or more drinks for females, is a significant public health concern [1, 2]. According to the World Health Organization's (WHO) Global Status Report on Alcohol and Health, in 2019 approximately 17% of individuals aged 15 years and older worldwide reported engaging in HED at least once in the past month [3]. For the United States (US), WHO reports higher prevalence, with 33.2% of adults engaging in HED in the past 30 days. This drinking pattern is strongly linked to an increased risk of acute and chronic outcomes [4, 5, 6, 7]. Acutely, HED increases the risk of injuries and alcohol poisoning by enhancing inhibitory neurotransmission, and impairing motor coordination and judgement [4]. Chronically, HED is associated with cardiovascular diseases such as hypertension and ischemic heart disease due to oxidative stress and inflammation. It increases the risk of liver diseases and impairs the immune system, making the body more susceptible to infections [6, 7]. Understanding and addressing HED is crucial for informing public health strategies and policies. HED is a key component in modelling the alcohol‐attributable burden of disease; however, many surveys exhibit missingness or inconsistencies in its reporting [8].

In such cases, predictive models offer a method to estimate HEDs' likelihood at the individual level. While logistic regression is commonly used, recent advancements in machine learning algorithms (MLA) have shown potential to outperform logistic regression in terms of predictive accuracy and robustness. MLAs are extensively used in medical science [9, 10, 11]. A systematic review by Mak et al. [12] identified that MLAs are increasingly used in addiction psychiatry for informing medical decisions. However, the limited number of studies included in this review (14 studies covered substance use, of which only 3 focused on alcohol use) may not adequately reflect the full potential of MLAs in alcohol research [13]. In recent years, other studies have surfaced which used MLAs to predict alcohol use disorders [11, 14], alcohol treatment outcomes [15] and alcohol use amongst certain populations [16, 17].

Logistic regression, while also being an MLA, is considered the traditional standard for prediction due to its interpretability and reliance on well‐defined statistical assumptions [18]. In contrast, other MLAs focus on directly learning patterns from data and can automatically capture complex, non‐linear relationships. This flexibility makes MLAs particularly useful for predictive modelling, though it often requires specific techniques to prevent overfitting [19]. These models can be integrated into public health surveillance systems to support the imputation of HED risk when direct indicators are unavailable, ultimately improving the accuracy of burden of disease estimates and guiding targeted prevention strategies. In addition, such models can be applied to predict HED at the individual level in simulation models. Beyond alcohol use, similar methodologies could be adapted to predict acute patterns of other substances, such as cannabis, further demonstrating the flexibility and applicability of these models in various public health contexts.

Given these advantages, our goal was to compare six supervised MLAs, including logistic regression, which analyzed 11 features, and to determine the most suitable model for predicting HED within the US population using nationally representative survey data. By concentrating on a reduced set of relevant variables, we aimed to identify which variables serve as the most influential predictors for HED and to create models that are both interpretable and practical for real‐world applications [13].

## 2 Methods

### 2.1 Data Source

Participant demographics and health behaviours data were extracted from the 1997 to 2018 National Health Interview Survey (NHIS) [20]. The NHIS is an annual, nationally representative, cross‐sectional survey that collects health information on the civilian non‐institutionalised population residing in the 50 states and the District of Columbia in the US. It uses a complex multistage probability sampling design to select participants. Its data collection, conducted through face‐to‐face interviews, minimises biases. NHIS also provides a large sample size and includes detailed questions on alcohol consumption, distinguishing between lifetime abstainers and former drinkers. These factors make it particularly well‐suited for selecting our target population. Data were compiled and distributed by IPUMS Health Surveys (http://www.nhis.ipums.org), an integrated dataset based on the NHIS public use files [21].

**FIGURE 1:**
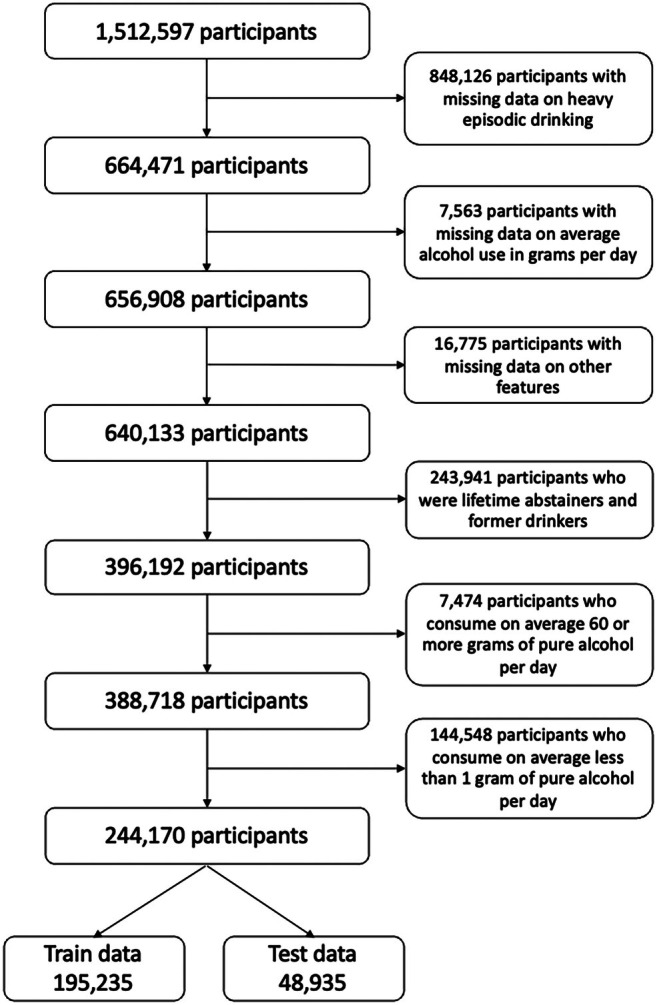
Selection of our study population and data split.

The target population was the US adult general population (i.e., 18 years old or older). After excluding participants with missing data on our key variables, we excluded participants who reported being lifetime abstainers and former drinkers under the premise that they do not engage in HED. Similarly, we excluded participants who reported drinking 60 g of pure alcohol per day or more on average, under the premise that they are engaging in heavy daily drinking. Finally, we excluded participants who reported drinking less than 1 g of pure alcohol per day, to minimise potential misclassification amongst these individuals as current drinkers. Figure 1 summarises our selection criteria.

We divided our dataset into two portions: 80% of all observations were allocated to the training dataset, while the remaining 20% constituted the test dataset. Subsequently, each model was exclusively trained and cross‐validated on the training dataset, then evaluated using the test dataset [22].

### 2.2 Predictors and Outcome

In all, 11 variables were selected as predictors in this study: average daily alcohol use, age, sex, educational attainment and family income (socioeconomic status indicators), race and ethnicity, smoking status, marital status, psychological distress, health status and region of residence. They were selected due to their established relationships with alcohol consumption behaviours and their influence on health outcomes. Average daily alcohol use and age are direct predictors of drinking patterns, while sex, socioeconomic status, race and ethnicity are important demographic factors that are known to modify drinking behaviours and their associated risks [23, 24, 25, 26]. The inclusion of smoking status, marital status, psychological distress, health status and region further allows us to capture individual behavioural, mental health, social and geographic dimensions relevant to HED in the US population.

Participants provided information about their alcohol consumption patterns, including the quantity and frequency of alcohol intake over their lifetime and in the past year. We calculated daily grams of alcohol consumption using the following equation: (number of days alcohol was consumed in the past year/365) × (average number of drinks on days alcohol was consumed × 14 g/drink), assuming 14 g of pure alcohol per standard drink.

Age at the time of survey was used as a continuous variable. Region was categorised into Northeast, North Central/Midwest, South and West. The following variables were all self‐reported. Socioeconomic status was measured via educational attainment and income. Educational attainment was categorised into high education (individuals with a bachelor's degree or higher), medium education (some college) and low education (high school degree or less). Income was categorised into high income (≥ 400% of federal poverty threshold), middle income (200%–399% of federal poverty threshold), lower income (< 199% of federal poverty threshold), and a missing category. Race and ethnicity were classified as non‐Hispanic White participants (hereafter, White participants), non‐Hispanic Black participants (hereafter, Black participants), Hispanic participants and other participants (non‐Hispanic Asian and Pacific Islander, American Indian and Alaska Native, and non‐Hispanic all other race groups). Smoking status was categorised as never smoker, former smoker, current some‐day smoker, and current every‐day smoker. Marital status was classified as married or living with a partner versus never married, widowed, divorced, or separated. Psychological distress was assessed using the K6 scale and categorised as none to low (K6 score < 5), moderate (K6 score ≥ 5 and < 13) and severe (K6 score ≥ 13). Health status was based on self‐perceived health and classified as excellent, very good, good, fair or poor.

Our outcome was HED (participants reported the frequency of HED in the past year, defined as consuming 5 or more (for men) or 4 or more (for women) standard drinks in a single day) which was operationalised as a binary variable (engaging in HED at least once per month (and less than daily) or not). Given the conceptual overlap between average daily alcohol consumption and HED, we assessed their relationship by calculating the point‐biserial correlation. The correlation coefficient was *r* = 0.48, indicating a moderate association. This suggests that while related, these measures capture distinct patterns of alcohol use and provide complementary information.

### 2.3 Model Selection

Six MLA were trained and evaluated to determine the most appropriate model for predicting HED within the US population (for definitions, see Table e1): two probabilistic models (logistic regression and naïve Bayes), a distance‐based algorithm (K‐Nearest Neighbours, kNN), a support vector machine—radial model, and two ensemble methods (random forest and extreme gradient boosting (XGBoost)). These classification methods were chosen based on prior literature and due to their strengths and limitations [12, 13, 14]. While all other models' algorithms inherently capture interaction effects, we manually tested for all possible two‐way interactions in the logistic regression. We conducted hyperparameter tuning using grid search [27, 28]. This method systematically explores a predefined range of hyperparameters (i.e., model‐specific settings that are not learned from the data but must be defined prior to training), assesses the performance of each set by evaluating the cost function and identifies the set that delivers the best performance according to the selected accuracy metric. For grid search strategy and final hyperparameter values selected see Table e1. During the model‐training phase, 10‐fold cross‐validation was performed to optimise the tuning parameters [29].

### 2.4 Model Evaluation

The best performance models found after cross‐validation with grid search were evaluated on the ‘test’ dataset. A 0.5 probability threshold was applied to models that output class probabilities, whereas models like kNN and support vector machine used their default class assignment mechanisms (i.e., majority vote for kNN and decision boundaries for support vector machine). The following values were calculated to measure each algorithm's predictive performance [13, 30]. Accuracy reflects the overall correctness of the model by showing the proportion of correct predictions. Recall, or sensitivity, highlights the model's ability to identify positive cases when they are truly present. Specificity measures how well the model detects negative cases, correctly identifying when a condition is absent. Precision (i.e., positive predictive value) indicates the reliability of positive predictions, showing how often the model is right when it predicts a positive outcome. Negative Predictive Value (NPV) reveals the accuracy of negative predictions, indicating how often a negative prediction is correct. F1 prediction score, is defined as the balance between the precision and the recall. Kappa measures inter‐rater agreement, comparing the observed accuracy with the expected accuracy if the ratings were random. Lastly, we also calculated the area under the curve (AUC) of the receiver‐operating characteristic, which reflects how well the model can distinguish between individuals with and without the outcome. A higher AUC indicates better performance in correctly ranking those at higher risk above those at lower risk.

### 2.5 Imbalanced Data Handling

The dataset exhibited class imbalance, with 78.7% of observations classified as non‐HED and 21.3% as HED. To address this issue, two different strategies were tested separately. For the first strategy, we created a balanced training dataset by randomly sampling equal proportions of HED and non‐HED cases, ensuring that both classes were equally represented during model training (train data *n* = 83,222). The test dataset remained the same. In the second strategy, class weights were introduced to further enhance their sensitivity to the minority class, ensuring a more balanced consideration in the predictive modelling process. Class weights were only implemented for logistic regression and XGBoost using the caret framework, as the underlying implementation does not support observation weights for the other models.

### 2.6 Model Interpretability and Feature Importance

Feature importance for the highest‐performing model compared to the other models was evaluated using Shapley Additive Explanations (SHAP) [31]. SHAP values offer a unified measure of feature importance by quantifying the marginal contribution of each feature to the model's predictions. We focused on global feature importance by aggregating the absolute SHAP values across all observations in the test dataset. This approach provides an overall ranking of each feature's contribution to the model's ability to distinguish between HED‐positive and HED‐negative cases across the entire population. Summary plots were generated to visualise the distribution of SHAP values across all features, while feature dependence plots illustrated how variations in individual feature values influence the predicted likelihood of the outcome [32].

### 2.7 Secondary Analysis

In a secondary analysis, we evaluated the performance of the models in predicting HED amongst individuals with varying levels of average daily alcohol consumption. Specifically, we tested the models on two distinct subsets of the test data: participants consuming 1–5 g of alcohol per day and those consuming 40–60 g per day. This analysis aimed to assess how well the selected model performs in predicting HED in populations at the lower and upper ends of alcohol consumption.

Model development and reporting was conducted according to the TRIPOD guidelines [33]. All models were developed, validated and evaluated using the *caret* package, and SHAP analysis was done using the *SHAPforxgboost* package in R software version 4.4.1 [31, 34, 35].

## 3 Results

Our final dataset included a total of 244,170 participants (43.4% women), with 195,235 participants in the training dataset and 48,935 participants in the test dataset. Table 1 displays the sample characteristics for both the training and test datasets by HED.

**TABLE 1:** Descriptive information of National Health Interview Survey train and test dataset.

	No HED	HED
Total sample size, n	192,195	51,975
Female, *n* (%)	90,933 (47.31)	15,019 (28.90)
Age, mean (SD)	46.0 (16.7)	37.4 (13.8)
Alcohol use, mean grams/day (SD)	8.2 (8.4)	21.4 (14.2)
Alcohol use, *n* (%)		
≥ 1 to ≤ 20 g/day	176,618 (91.90)	29,061 (55.91)
> 20 to ≤ 40 g/day	12,713 (6.61)	16,813 (32.35)
> 40 to ≤ 60 g/day	2864 (1.49)	6101 (11.74)
Education, *n* (%)		
Low (High school)	61,458 (31.98)	21,488 (41.34)
Medium (Some college)	59,083 (30.74)	17,592 (33.85)
High (Bachelors)	71,654 (37.28)	12,895 (24.81)
Income, *n* (%)		
Lower	16,817 (8.75)	7553 (14.53)
Middle	69,367 (36.09)	22,464 (43.22)
High	76,113 (39.60)	16,586 (31.91)
Missing	29,898 (15.56)	5372 (10.34)
Race and ethnicity, *n* (%)		
White participants	160,831 (83.68)	44,146 (84.94)
Black participants	21,348 (11.11)	4743 (9.13)
Hispanic participants	2288 (1.19)	1115 (2.15)
Other	7728 (4.02)	1971 (3.79)
Smoking status, *n* (%)		
Never smoker	99,388 (51.71)	20,315 (39.09)
Former smoker	51,199 (26.64)	10,886 (20.94)
Current someday smoker	10,489 (5.46)	5665 (10.90)
Current every day smoker	31,119 (16.19)	15,109 (29.07)
Married status, *n* (%)		
Not married/cohabitating	98,079 (51.03)	33,908 (65.24)
Married/cohabitating	94,116 (48.97)	18,067 (34.76)
Psychological distress, *n* (%)		
None/low	159,562 (83.02)	39,823 (76.62)
Moderate	28,220 (14.68)	10,298 (19.81)
Severe	4413 (2.30)	1854 (3.57)
Health status, *n* (%)		
Excellent	65,557 (34.11)	16,499 (31.74)
Very good	69,432 (36.13)	18,600 (35.79)
Good	42,991 (22.37)	12,788 (24.60)
Fair	11,644 (6.06)	3387 (6.52)
Poor	2571 (1.34)	701 (1.35)
Region, *n* (%)		
Northeast	37,243 (19.38)	8198 (15.77)
North Central/Midwest	44,778 (23.30)	14,339 (27.59)
South	62,094 (32.31)	17,384 (33.45)
West	48,080 (25.02)	12,054 (23.19)

Abbreviations: HED, heavy episodic drinking; SD, standard deviation.

**FIGURE 2:**
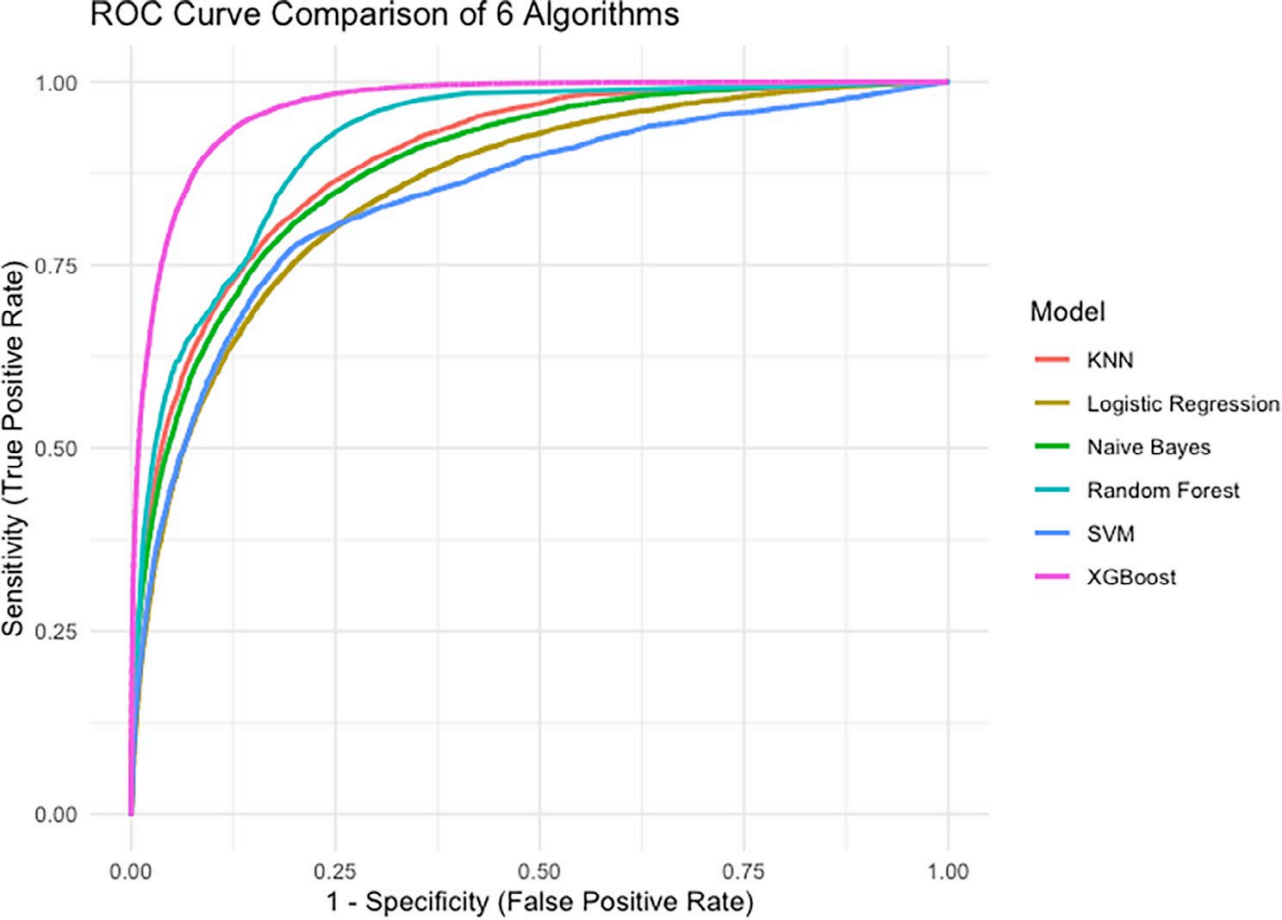
Receiver operating characteristic (ROC) curves of all the models.

Table 2 presents the prediction performance indices on the test set for the six algorithms, after training with cross‐validation on the train set. The AUCs for the MLA varied. All models demonstrated strong discriminative ability. This indicates that for these models, the probability of correctly ranking a randomly selected HED instance higher than a non‐HED instance ranged from 0.85 to 0.97 (excellent ability). XGBoost outperformed the other models, with a sensitivity of 0.80, a precision of 0.83 and an accuracy of 0.92. The receiver‐operating characteristic curves are displayed in Figure 2.

**TABLE 2:** Model performance metrics.

	Accuracy (95% CI)	Sensitivity	Specificity	PPV	NPV	F1 score	Kappa	AUC (95% CI)
Logistic regression	0.846 (0.843–0.849)	0.442	0.955	0.724	0.864	0.549	0.462	0.866 (0.862–0.87)
Naïve Bayes	0.855 (0.851–0.858)	0.509	0.948	0.724	0.878	0.598	0.513	0.885 (0.882–0.889)
kNN	0.867 (0.864–0.870)	0.539	0.956	0.766	0.885	0.632	0.554	0.891 (0.887–0.894)
SVM	0.845 (0.842–0.848)	0.396	0.966	0.756	0.856	0.519	0.438	0.849 (0.844–0.853)
Random forest	0.876 (0.873–0.879)	0.604	0.949	0.761	0.899	0.673	0.598	0.917 (0.914–0.920)
XGBoost	0.923 (0.921–0.926)	0.796	0.958	0.834	0.946	0.815	0.766	0.970 (0.969–0.972)

Abbreviations: AUC, area under the curve; CI, confidence interval; kNN, k‐nearest neighbour; NPV, negative predictive value; PPV, positive predictive value; SVM, support vector machine; XGBoost: extreme gradient boosting.

In addressing the class imbalance within the dataset, both separate strategies yielded similar results, improving models' ability to predict the minority class (Table e2). The models showed noticeable increases in sensitivity while maintaining overall accuracy, though some loss in precision was observed.

**FIGURE 3:**
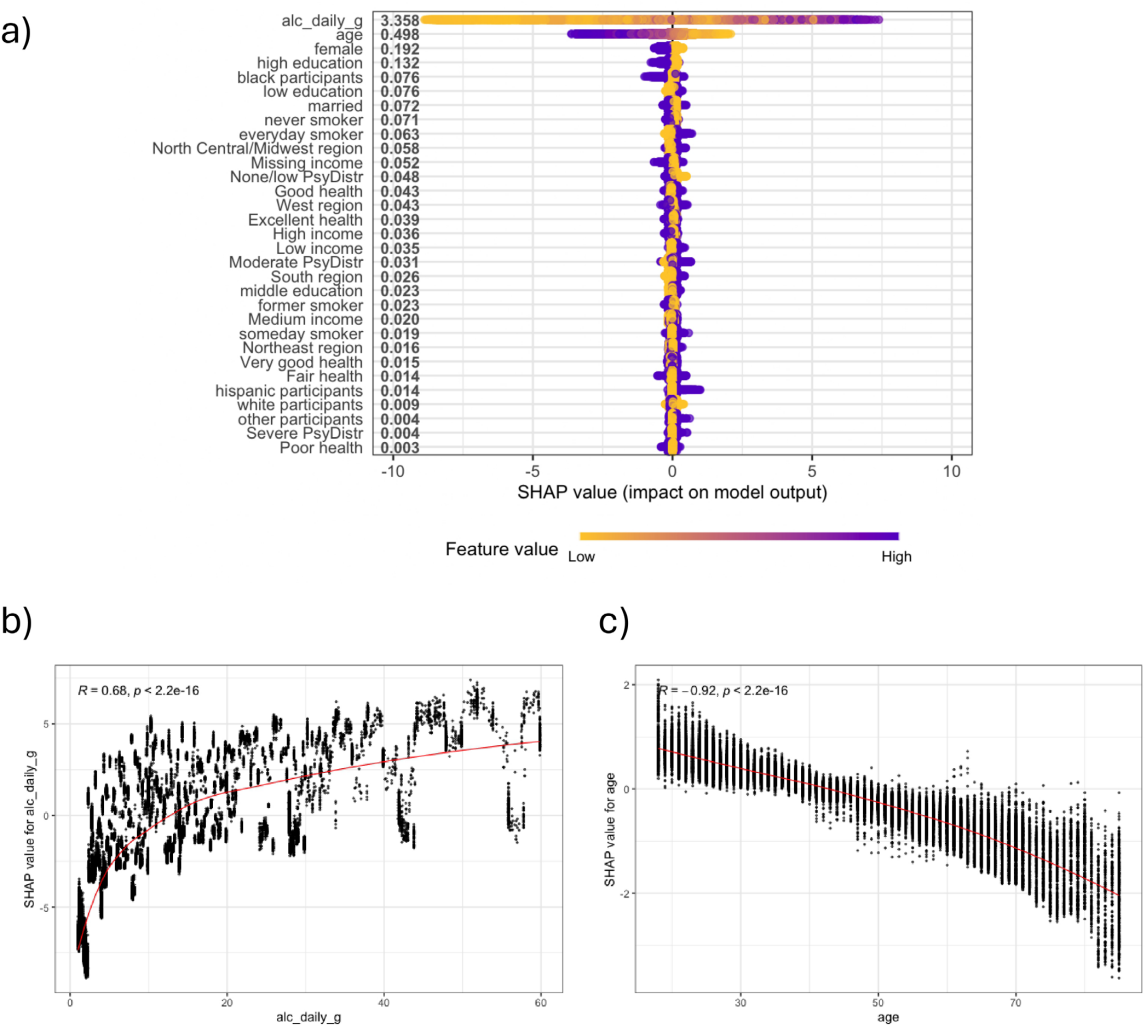
(a) SHAP analysis of the XGBoost model: The horizontal position indicates whether the effect of that value is associated with a higher or lower prediction, while the colour represents whether the variable is high (in purple) or low (in yellow) for that observation. (b) SHAP dependence plot of the relationship between the feature average daily alcohol consumption in grams and its SHAP values. (c) SHAP dependence plot of the relationship between the feature age and its SHAP values.

To assess feature importance and interpretability in XGBoost, SHAP values were calculated. The SHAP summary plot (Figure 3a) displayed the global feature importance, calculated by aggregating the absolute SHAP values across all observations, with alcohol use and age being the most influential factors in classifying HED. The feature importance plot can be found in Appendix Figure e1. The SHAP dependence plot illustrated how the model differentiates between HED and non‐HED cases: higher alcohol consumption contributed to an increased predicted probability of HED, whereas lower alcohol consumption contributed to a decreased predicted probability (Figure 3b). Similarly, younger age had a stronger positive contribution to the predicted probability of HED, while older age contributed negatively to the prediction (Figure 3c).

In a secondary analysis, the XGBoost model's performance was evaluated on individuals with alcohol consumption at the lower (1–5 g/day, *n* = 20,439, HED = 4% vs. no HED = 96%) and higher (40–60 g/day, *n* = 1827, HED = 67% vs. no HED = 33%) ends of the spectrum. The model demonstrated strong predictive ability for both groups, with a notably high AUC and accuracy in detecting HED amongst the lowest consumption group (Table e3). However, in the lower spectrum, the model struggled with precision.

## 4 Discussion

Our study implemented six MLAs to compare their performance in predicting HED and to identify the features that most influence this prediction. Amongst these models, XGBoost stood out, demonstrating strong predictive accuracy compared to the other MLAs we trained. This finding also underscores the advantages of MLAs over traditional methods like logistic regression. MLAs can provide more detailed predictions, making them particularly valuable for modelling health behaviors like HED that may be influenced by multiple interacting factors.

Previous studies have used similar methodologies. Afzali et al. [16] compared seven algorithms using a cross‐cultural, cross‐study scheme to predict different levels of alcohol use in mid‐adolescence, finding that the elastic‐net algorithm showed the best performance. Bonnell et al. [36], using data from the 1996–2016 National Health and Nutrition Examination Survey, developed a decision tool to aid in screening for unhealthy alcohol use (defined as ≥ 1 drink per day for women or ≥ 2 for men or binge drinking ≥ 1 per month in the past 12 months) and found that the random forest algorithm outperformed logistic regression in classification accuracy. Finally, Dell et al. [17] assessed the predictive power of three algorithms—logistic regression, classification trees, and random forest—finding that random forest had the highest performance in accurately classifying binge drinking (defined as a binary event occurring in the past month) amongst young adults aged 18–25 using data from the 2015–2018 National Survey on Drug Use and Health. Our study identified an ensemble method as having superior performance compared to the rest of our tested models and expanded on previous research by using a larger survey sample of adults 18 and older, incorporating age as a feature, evaluating more algorithms, and using SHAP analysis to identify key features. While other studies used a broader set of features, we focused on key predictors to enhance interpretability and practical applicability. This approach ensures the models remain manageable and actionable for public health interventions while providing robust predictions. Our models performed competitively, suggesting that a carefully chosen set of relevant variables can yield reliable and insightful predictions. Finally, further evaluation revealed that when class imbalance is properly accounted for (i.e., through resampling strategies or class weighting) the sensitivity of the models, particularly XGBoost, improves. This may be related to the inherent strength of boosting algorithms in minimising false negatives by sequentially focusing on misclassified cases, which enhances their sensitivity relative to other models.

A key advantage of machine learning algorithms is their ability to rank variables by influence, offering a clearer understanding of each feature's impact on predictions [32, 37, 38]. Using SHAP to explain the XGBoost model, we identified that average daily alcohol use was the most influential predictor variable, followed by age. Our results align with existing alcohol research, which finds that higher alcohol consumption and younger age are strong predictors of HED risk [39]. SHAP values provide insights into the model's classification process by quantifying the contribution of each predictor to the final decision [40]. Implementing a SHAP analysis is particularly valuable in machine learning applications, as it allows for a deeper understanding of how complex models make predictions, ensuring transparency and potential applicability in public health interventions. The secondary analysis showed that the model maintained strong predictive ability for both low and high alcohol consumption groups, with particularly high accuracy amongst the lowest consumption group. However, precision was lower for this side of the spectrum. This finding is expected, as HED is relatively rare amongst individuals who typically consume minimal amounts of alcohol on average. In such low‐prevalence settings, even a small number of false positives can substantially lower precision, despite good model discrimination. This also aligns with our SHAP findings and reflects on a key distinction: SHAP values explain which features drive individual predictions, whereas model performance metrics assess how well the model generalises across subgroups. These findings highlight the importance of considering both interpretability (via SHAP) and model performance metrics when evaluating MLA results [31].

### 4.1 Strengths and Limitations

Our study presents several strengths. First, our study counted on a large sample size and careful attention was given to addressing overall class imbalance, a common challenge in predictive modelling, ensuring that the model did not disproportionately favour the majority class. Additionally, our study incorporated a traditional classification method, logistic regression, alongside more advanced MLA. This inclusion provides valuable context by highlighting the comparative advantages of other MLA, such as their ability to capture complex, non‐linear relationships and improve predictive performance. In contrast to logistic regression (where interaction terms must be explicitly specified and retained based on subjective or clinical judgement) MLA offers a more data‐driven approach, automatically identifying relevant interactions without requiring manual input or assumptions about variable relationships. By incorporating SHAP, our study provides transparent model interpretability, making it easier to understand how individual features impact HED predictions [38]. Finally, the model's robustness was tested against extreme values of alcohol use, further validating the model's capacity to handle a wide spectrum of input data.

There were several limitations in our study. Our dataset relied on self‐reported alcohol consumption, which may be subject to biases, leading participants to underreport behaviours perceived negatively, and is likely skewed by the underrepresentation of heavier drinkers. Additionally, we defined HED as a binary variable and did not consider other frequencies of HED. Future studies should explore algorithms capable of predicting categorical outcomes while incorporating more nuanced measures of drinking frequency. For example, deep learning models could predict multi‐level risk categories using detailed HED frequency data, while unsupervised clustering techniques could identify distinct drinking behaviour profiles that better capture real‐world variability and inform outcome prediction. Another limitation is the lack of detailed geographic variables in our dataset, which is based on the public access NHIS data; however, we included region as a feature in our models to partially account for geographic variation. Finally, we did not validate our models using a completely independent dataset. Instead, we relied on a subset of the same dataset for testing. This highlights a key limitation—MLA can only be as accurate as the data it replicates. External validation remains challenging, as available objective aggregate measures, such as alcohol per capita consumption, do not directly map onto individual‐level HED prevalence. Future research should focus on integrating objective sources for external validation, such as biomarker data or linked administrative records, to strengthen model reliability and applicability.

### 4.2 Practical Applications

Beyond methodological advancements, predictive models like ours have practical applications. Organisations such as WHO frequently rely on statistical models to estimate global alcohol consumption patterns, and integrating machine learning‐driven predictions could improve the accuracy of such estimates by imputing likely cases in datasets where direct measurement is absent, supporting accurate population‐level estimates and resource planning. Additionally, complex policy modelling frameworks could benefit from incorporating MLA to dynamically estimate individual‐level HED prevalence in synthetic cohorts within simulation models, enhancing data‐driven decision‐making. While XGBoost performed best in our study, the optimal choice of model may vary depending on dataset characteristics, emphasising the importance of a comparative approach in future research. We recommend that future studies prioritise evaluating the top‐performing models identified here, particularly in diverse populations and datasets. These algorithms could also be implemented in the context of other substance use, such as cannabis consumption, which may present similar challenges related to self‐reporting bias and underestimation of use. Given these limitations, applying machine learning models like the ones we compared could enhance the classification of high‐risk substance users by identifying subtle patterns and predictors that traditional methods may overlook. Improving data quality remains a critical step in refining predictive models and MLAs could be leveraged to detect and adjust for systematic biases.

## 5 Conclusions

In conclusion, our findings highlight the potential of MLAs to outperform traditional epidemiological methods in alcohol research, underscoring an opportunity for a shift towards more advanced modelling approaches in this field. As MLAs continue to evolve, understanding the mechanisms and key variables driving their predictions will be crucial for guiding effective interventions and supporting data‐driven decision‐making in public health.

## Author Contributions

**Laura Llamosas‐Falcón and Jürgen Rehm:** conceptualisation. **Laura Llamosas‐Falcón, Erik Spence and Jürgen Rehm:** methodology. **Laura Llamosas‐Falcón:** data curation. **Laura Llamosas‐Falcón:** formal analysis. **Charlotte Probst:** funding acquisition. **Laura Llamosas‐Falcón, Charlotte Probst, Kevin Shield, Erik Spence, Jürgen Rehm:** investigation. **Charlotte Probst and Jürgen Rehm:** project administration. **Charlotte Probst and Jürgen Rehm:** resources. **Charlotte Probst and Jürgen Rehm:** supervision. **Erik Spence and Jürgen Rehm:** validation. **Laura Llamosas‐Falcón, and Jürgen Rehm:** writing – original draft. **Laura Llamosas‐Falcón, Charlotte Probst, Kevin Shield, Erik Spence, Jürgen Rehm:** writing – review and editing. All authors have read and agreed to the published version of the manuscript.

## Ethics Statement

All adult participants in the NHIS provided written informed consent. Data collection for NHIS was approved by the Research Ethics Review Board of the National Center for Health Statistics (NCHS) and the US Office of Management and Budget.

## Consent

Patients or the public were not involved in the design, conduct, reporting, or dissemination plans of our research.

## Conflicts of Interest

The authors declare no conflicts of interest.

## Supporting information


**Table e1.** Grid search for six machine learning algorithms and the final hyperparameters selected.
**Table e2**. Results of model performance following class imbalance mitigation approaches.
**Figure e1**. Feature importance plot from the XGBoost model.
**Table e3**. Model performance across low and high daily average alcohol use using XGBoost model.

## Data Availability

The data that support the findings of this study are available in IPUMS at https://nhis.ipums.org/nhis/. These data were derived from the following resources available in the public domain: IPUMS NHIS Get Data, https://nhis.ipums.org/nhis‐action/variables/group.
