# Supplementary material for: Machine Learning Algorithms to Predict Heavy Episodic Drinking in the United States Using Survey Data
Source: Drug Alcohol Rev. 2025 Nov 4;45(1):e70065. doi: 10.1111/dar.70065 (PMC12682420; doi:10.1111/dar.70065)
Supplement: Supplementary file 1 — Table e1. Grid search for six machine learning algorithms and the final hyperparameters selected. Table e2. Results of model performance following class imbalance mitigation approaches. Figure e1. Feature importance plot from the XGBoost model. Table e3. Model performance across low and high daily average alcohol use using XGBoost model. [file DAR-45-0-s001.docx]

### Appendix

**Table e1**. Grid search for six machine learning algorithms and the final hyperparameters selected

| **Model** | **Definition** | **Grid search** | **Hyperparameters selected** |
| --- | --- | --- | --- |
| Logistic regression^1^ | Logistic regression is a fundamental machine learning algorithm used for binary classification tasks. It models the probability of a binary outcome based on one or more predictor variables using the sigmoid function, which maps input features to probabilities. The algorithm calculates a weighted sum of the input features, applies the sigmoid function to this sum, and uses a threshold (commonly 0.5) to classify the data points. Assumptions include linearity of the features, independence of observations, and no multicollinearity. | – | – |
| Naïve Bayes^2^ | Naïve Bayes uses a simplistic probabilistic approach based on the Bayes theorem to determine the value with the highest probability calculated from a chain of conditional probabilities. It is called “naïve” because it assumes that the features being evaluated are independent of each other. | **usekernel = c(TRUE, FALSE)**: This determines whether a kernel density estimator is used to approximate the distribution of continuous features instead of assuming a normal distribution.  **fL = c(0, 0.5, 1)**: Laplace smoothing parameter helps handle cases where some feature values do not appear in the training data.  **adjust = c(0.5, 0.75, 1, 1.25, 1.5)**: This parameter adjusts the bandwidth of the kernel density estimator when usekernel = TRUE. | The final values used for the model were fL = 0.5, usekernel = TRUE and adjust = 0.75. |
| kNN^3^ | kNN is a non-parametric classification algorithm. It identifies the K nearest data points by computing the distance between a new data point (i.e. data not included in the training set) and those in the training set. Subsequently, the algorithm assigns the new data point to the class that most of its K nearest neighbors belong to, using a majority voting mechanism. | **k = seq(5, 63, 2)**: This defines the number of neighbors the algorithm considers when classifying a new data point. | The final value used for the model was k = 37. |
| SVM^4^ | SVM algorithms aim to classify data points by finding a boundary, called a *hyperplane*, that best separates the data into different categories. SVMs place this hyperplane so that the distance between the hyperplane and the closest data points from each category is maximised. These closest data points, which influence the placement and orientation of the hyperplane, are called *support vectors*. We implemented a radial model. | **C = c(0.1, 1, 5)**: controls the trade-off between maximising the margin and minimising classification errors.  Sigma was set constant at 0.01 | The final value used for the model was C = 5. |
| Random forest^5^ | Random Forest is a model that combines multiple decision trees to classify data points. From randomly selected subsets of training data, random forest generates a set of decision trees, and then it specifies the class label of a data point by aggregating the votes from different decision trees based on a majority vote. The random forest is more powerful than a single decision tree classifier because it avoids overfitting on the training data. | **mtry = 2:6**: controls how many predictors (or features) are randomly selected at each split in the decision trees. | The final value used for the model was mtry = 6. |
| XGBoost^6^ | XGBoost builds a strong predictive model by sequentially adding decision trees, where each new tree corrects the errors of the previous ones. The individual decision trees, known as "weak learners," typically have high bias and are only slightly better than random guessing. However, when combined through the boosting process, these weak learners contribute valuable insights, collectively reducing both bias and variance to create a much stronger, more accurate predictive model. XGBoost is known for its ability to handle large datasets efficiently and prevent overfitting through regularisation techniques. | First, we fixed the number of iterations (**nrounds = c(200, 300, 400, 500, 600)**) and used a relatively high learning rate (**eta = c(0.05, 0.1, 0.3)**). Next, we adjusted the parameters “max_depth” (**3 ± 1, or +2 if max_depth = 2**) and “min_child_weight” (**c(1, 2, 3)**) for the selected learning rate. Then, we experimented with different values for row and column sampling. After determining the best values from this step, we optimised the gamma parameter (**c(0, 0.05, 0.1, 0.5, 0.7, 0.9, 1.0)**). Finally, the learning rate was reduced (**eta = c(0.01, 0.015, 0.025, 0.05, 0.1)**) to fine-tune the model. | The final values used for the model were: nrounds = 600; eta = 0.1; max_depth= 5; min_child_weight = 1; gamma = 0.05; colsample_bytree = 0.8; subsample= 1 |

**Table e2**. Results of model performance following class imbalance mitigation approaches

|  | Accuracy (95% CI) | Sensitivity | Specificity | PPV | NPV | F1 score | Kappa | AUC (95% CI) |
| --- | --- | --- | --- | --- | --- | --- | --- | --- |
| **Class = HED** | | | | | | | | |
| Downsampling |  |  |  |  |  |  |  |  |
| Logistic regression | 0.802 (0.799-0.806) | 0.756 | 0.814 | 0.523 | 0.926 | 0.618 | 0.490 | 0.867 (0.864-0.871) |
| Naïve Bayes | 0.812 (0.809-0.816) | 0.776 | 0.822 | 0.539 | 0.932 | 0.637 | 0.515 | 0.885 (0.881-0.888) |
| kNN | 0.814 (0.811-0.818) | 0.794 | 0.820 | 0.542 | 0.937 | 0.644 | 0.525 | 0.891 (0.888-0.895) |
| SVM | 0.796 (0.793-0.800) | 0.782 | 0.800 | 0.512 | 0.932 | 0.619 | 0.488 | 0.872 (0.868-0.875) |
| Random Forest | 0.823 (0.819-0.826) | 0.856 | 0.814 | 0.553 | 0.955 | 0.672 | 0.558 | 0.912 (0.915-0.920) |
| XGBoost | 0.897 (0.894-0.900) | 0.932 | 0.887 | 0.690 | 0.980 | 0.793 | 0.726 | 0.971 (0.971-0.972) |
| Class weighting |  |  |  |  |  |  |  |  |
| Logistic regression | 0.804 (0.800-0.807) | 0.758 | 0.816 | 0.525 | 0.926 | 0.620 | 0.494 | 0.868 (0.864-0.872) |
| XGBoost | 0.899 (0.896-0.902) | 0.931 | 0.890 | 0.695 | 0.980 | 0.796 | 0.731 | 0.971 (0.969-0.972) |
| Class = No HED | | | | | | | | |
| Downsampling |  |  |  |  |  |  |  |  |
| Logistic regression | 0.802 (0.799-0.806) | 0.814 | 0.756 | 0.926 | 0.523 | 0.866 | 0.490 | 0.867 (0.864-0.871) |
| Naïve Bayes | 0.812 (0.809-0.816) | 0.822 | 0.776 | 0.932 | 0.539 | 0.873 | 0.515 | 0.885 (0.881-0.888) |
| kNN | 0.814 (0.811-0.818) | 0.820 | 0.794 | 0.937 | 0.542 | 0.874 | 0.525 | 0.891 (0.888-0.895) |
| SVM | 0.796 (0.793-0.800) | 0.800 | 0.782 | 0.932 | 0.512 | 0.861 | 0.488 | 0.872 (0.868-0.875) |
| Random Forest | 0.823 (0.819-0.826) | 0.814 | 0.856 | 0.955 | 0.553 | 0.879 | 0.558 | 0.912 (0.915-0.920) |
| XGBoost | 0.897 (0.894-0.900) | 0.887 | 0.932 | 0.980 | 0.690 | 0.931 | 0.726 | 0.971 (0.971-0.972) |
| Class weighting |  |  |  |  |  |  |  |  |
| Logistic regression | 0.804 (0.800-0.807) | 0.816 | 0.758 | 0.926 | 0.525 | 0.868 | 0.494 | 0.868 (0.864-0.872) |
| XGBoost | 0.899 (0.896-0.902) | 0.890 | 0.931 | 0.980 | 0.695 | 0.933 | 0.731 | 0.971 (0.969-0.972) |

AUC, area under the curve; CI, confidence interval; NPV, negative predictive value; PPV, positive predictive value.

**
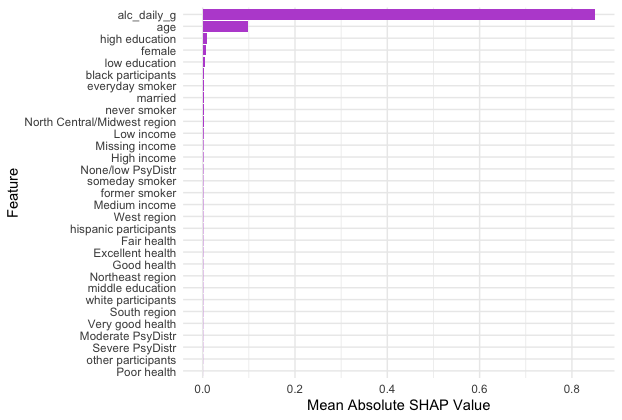
**

**Figure e1.** Feature importance plot from the XGBoost model.

SHAP, SHapley Additive exPlanations.

**Table e3**. Model performance across low and high daily average alcohol use using XGBoost model.

|  | Accuracy (95% CI) | Sensitivity | Specificity | PPV | NPV | F1 score | Kappa | AUC (95% CI) |
| --- | --- | --- | --- | --- | --- | --- | --- | --- |
| **Alc 1 - 5 gr/day** | 0.948 (0.945-0.951) | 0.758 | 0.957 | 0.454 | 0.758 | 0.568 | 0.543 | 0.971 (0.968-0.974) |
| **Alc 40 -** **60 gr/day** | 0.902 (0.887-0.915) | 0.979 | 0.747 | 0.886 | 0.946 | 0.930 | 0.766 | 0.962 (0.952-0.971) |

AUC, area under the curve; CI, confidence interval; PPV, positive predictive value; NPV, negative predictive value.

**References**

1. Harrell FE. *Regression Modeling Strategies: with applications to linear models, logistic regression, and survival analysis.* . New York: Springer-Verlag; 2010.

2. Rish I. An Empirical Study of the Naïve Bayes Classifier. *IJCAI 2001 Work Empir Methods Artif Intell*. 01/01 2001;3

3. Altman NS. An Introduction to Kernel and Nearest-Neighbor Nonparametric Regression. *The American Statistician*. 1992/08/01 1992;46(3):175-185. doi:10.1080/00031305.1992.10475879

4. Brereton RG, Lloyd GR. Support Vector Machines for classification and regression. 10.1039/B918972F. *Analyst*. 2010;135(2):230-267. doi:10.1039/B918972F

5. Breiman L. Random Forests. *Machine Learning*. 2001/10/01 2001;45(1):5-32. doi:10.1023/A:1010933404324

6. Chen T, Guestrin C. XGBoost: A Scalable Tree Boosting System. ACM; 2016:785-794.
